# Supplementary material for: A liver–heart endocrine axis revealed by systems genetics and mediated by hepatocyte growth factor activator
Source: medRxiv. 2026 May 6:2026.05.05.26352474. Preprint. [Version 1] doi: 10.64898/2026.05.05.26352474 (PMC13174734; doi:10.64898/2026.05.05.26352474)
Supplement: Supplement 1 [file NIHPP2026.05.05.26352474v1-supplement-1.pdf]

medRxiv preprint doi: https://doi.org/10.64898/2026.05.05.26352474; this version posted May 6, 2026. The copyright holder for this preprint (which was not certified by peer review) is the author/funder, who has granted medRxiv a license to display the preprint in perpetuity.
It is made available under a CC-BY 4.0 International license .

Supplemental Table 1

Baseline characteristics of subjects in Cleveland cohort

Characteristics	All Subjects (N=406)	Subjects without HFpEF (N=203)	Subjects with HFpEF (N=203)	P
Age, years	68.9 (60.8- 74.8)	68.7 (60.7- 74.4)	69.0 (60.8- 74.9)	0.82
Male sex (%)	44.8	44.3	45.3	0.92
Diabetes mellitus (%)	38.2	39.4	36.9	0.68
BMI (kg/m^2)	29.3 (25.0- 33.9)	29.1 (25.1- 33.2)	29.4 (25.0- 34.9)	0.45
Systolic blood pressure (mmHg)	135(121- 149)	136(122- 148)	130 (121- 150)	0.45
Hypertension (%)	72.1	65.7	78.5	<0.01
Current smoking (%)	8.4	7.4	9.4	0.58
CAD (%)	63.1	62.6	63.5	0.92
CVD (%)	64.5	63.5	65.5	0.76
History of MI (%)	30.0	26.0	34.0	0.11
LDL cholesterol (mg/dL)	95.0 (79.0-114.0)	95.5 (81.0-113.0)	94.0 (76.8-114.5)	0.22
HDL cholesterol (mg/dL)	35.3 (28.4-42.8)	35.5 (29.5- 42.4)	34.9 (27.1-43.3)	0.19
Total cholesterol (mg/dL)	161.0 (139.9-187.9)	161.3 (142.0-186.6)	159.2 (137.2-189.4)	0.39
Triglycerides (mg/dL)	118.0 (86.0-172.0)	117.5 (86.3-170.8)	118.5 (86.0-177.0)	0.90
CRP (mg/L)	3.38 (1.37-7.49)	2.88 (1.24-7.13)	3.66 (1.78-8.09)	0.04
eGFR (mL/min/1.73m^2)	83.9 (66.8-94.3)	88.3 (72.8- 97.0)	78.6 (61.5-92.0)	<0.001
LVEF (%)	55 (55- 60)	60(55- 65)	55(55- 60)	<0.01
NT-proBNP (pg/ml)	345.2 (151.3-943.9)	227.6 (90.7-508.4)	702.9 (263.3-1624.5)	<0.001
Hs-TnT (ng/L)	14.0 (7.3- 22.9)	10.9 (5.6- 18.8)	17.0 (9.3- 28.9)	<0.001
Statins (%)	51.5	55.2	47.8	0.16
Aspirin (%)	66.3	76.4	56.2	<0.001
Anti-diabetic drugs (%)	21.9	19.7	24.1	0.34
ACE inhibitors (%)	49.5	41.4	57.6	0.001
Calcium channel blockers (%)	22.2	15.8	28.6	0.003
Diuretics (%)	37.7	16.3	59.1	<0.001
HGF (ng/mL)	1.96(0.74- 5.65)	1.46(0.60- 5.26)	2.50 (1.00- 6.64)	<0.01
HGFAC (μg/mL)	3.93 (3.32- 4.73)	3.94(3.31- 4.73)	3.92(3.34- 4.72)	>0.99

Continuous data are presented median (interquartile range), categorical variables are presented as %. Wilcoxon-rank sum test for continuous variables and χ2 test for categorical variables were used to examine the difference between groups.
